# Companies’ behavior in measuring the quality of financial reports: Pre- and post-pandemic research

**DOI:** 10.3389/fpsyg.2022.1005941

**Published:** 2022-12-19

**Authors:** Tatiana Dănescu, Roxana Maria Stejerean

**Affiliations:** ^1^Department of Economic Sciences, Faculty of Economics and Law, University of Medicine, Pharmacy, Science and Technology “George Emil Palade” of Targu Mures, Târgu Mures, Romania; ^2^Doctoral School of Accounting, “1 Decembrie 1918” University of Alba Iulia, Alba Iulia, Romania

**Keywords:** quality of financial reporting, information asymmetry, BVB, regulated market, AeRO

## Abstract

Since information is the foundation for decision-making by its users, it and the quality associated with it must be given particular importance in order to reduce uncertainties about how it is reported and interpreted and to increase its usefulness. Financial reporting is of relatively great significance to both those who provide it and those who use it, with accounting providing a wide range of sources of financial information, ensuring a high degree of credibility compared to other sources of information. The objective of the research is to highlight the behavior of companies in measuring the quality of issuers’ financial reporting to identify solutions for harmonizing the way financial information is presented with the needs of users. Two hypotheses were defined and tested for this purpose, with the research being segmented over three successive stages. The first stage consists in identifying the appropriate index to measure the quality of financial reporting. The second stage consists in gathering the data and obtaining the quality measurements, for testing the defined hypotheses. The third stage concerns in concluding the results obtained highlight the existing divergences between the period before the health crisis, but also the period immediately after the COVID-19, on the two markets of the Bucharest Stock Exchange (regulated market and AeRO market).

## Introduction

The financial theory is based on the assumption that financial markets work in perfect harmony. However, financial markets are far from this excellence. Since participants in the capital market cannot access information provided by issuers at the same time and to the same extent, they often face an asymmetry in the level of information. The production of high-quality financial information is a fundamental element in ensuring the efficient functioning of capital markets, as financial reporting provides the main source of information on an entity’s profitability and development over time (Habib et al., 2019). However, we would like to highlight one of the purposes of this research and the resulting conclusions, namely that it is necessary to give high importance to the process of identifying information asymmetry in capital markets, to increase user trust in the information made available by public entities. The presence of information asymmetry in capital markets is captured in the literature review chapter, based in particular on the literature that emerged with the onset of the COVID pandemic (2019–2021).

The optimal investment decision relies to a large extent on the quantity and quality of economic information presented by the entity, correlated with both reality and its market image. Those who provide the information presented in financial reports need to be aware of its impact on society and on the people who come into contact with it, as it can generate many changes in the attitudes of those who use it, whether or not they favor a decision. Therefore, the next step is to emphasize the need to quantify the quality of the financial reports on which users base their investment decisions.

Two hypotheses were defined and tested for this purpose, with the research being segmented over three successive stages. The first stage consists in identifying the appropriate index to measure the quality of financial reporting. To measure the quality of the financial reports of the 105 companies included in our sample, we used the quality measurement index developed by the Nijmegen Centre for Economics (NiCE). This index is based on the quality characteristics of financial information established by the International Accounting Standards Board (IASB), including relevance and accurate representation, comparability, verifiability, timeliness, and understandability.

The second stage consists in gathering the data and obtaining the quality measurements, for testing the defined hypotheses. The third stage concerns in concluding the results obtained highlight the existing divergences between the period before the health crisis, but also the period immediately after the COVID 19 the two markets of the Bucharest Stock Exchange (regulated market and AeRO market).

## Definition of the problem under investigation

### Information asymmetry in capital markets

Information asymmetry is a condition where differences in internal information acquisition will affect the future of the relationship between management and investors. In this regard, it is necessary to empirically capture the level of information asymmetry in the process of identifying opportunities offered by a company (Evodila, 2020). However, it is considered a difficult task as information asymmetry is not a directly observable phenomenon (Abad et al., 2017).

It has been pointed out that higher quality of financial reporting, contributes to reducing information asymmetry and therefore improving the efficiency with which money funds are placed in capital markets (Al-Sakini, 2019).

In particular, a negative association is found between the quality of financial reporting and the existence of diverging investor views and is consistent with the dynamics regarding stock market returns (Abdel-Meguid, 2019).

We find that entities whose financial reporting is of lower quality in the first place tend to exhibit lower future stock returns. Second, poorer financial reporting quality directly means lower useful information content. This, as Silva and Cerqueira (2021) also points out, leads to a reduction in the number of information investors share and leads them to rely more on individualistic assessments, conflicting beliefs, and information obtained from various private sources, fostering disagreements among existing users within stock markets.

We also believe that there is currently a high risk of distortion of financial statements and implicit influencing the opinion and vision of end users who base their investment decision on the information found in financial reports (Dănescu et al., 2021). In this regard, the quality of reports also concerns elements such as ethics and fairness of management.

### Quality of financial reporting

The quality of financial reporting has remained an issue of major concern to accounting professionals, regulators, and other users of financial information. This is because financial reporting has been and continues to be a primary means of communicating the results of transactions and events that occurred within an entity to various users (Mbobo and Ekpo, 2016). Also, part of the research approach is directed toward the study of the social and psychological side of accounting, although there is no widely accepted definition of behavioral accounting (Dănescu and Luminiţa, 2013).

Despite the multitude of research conducted over the past decades, there is no precise and universally accepted definition of the quality of accounting information reported in financial reports (Fuad, 2019). We consider a reasonable rationale for judging the quality of information to be that expressed by Budai et al. (2021), namely that quality can be achieved when it influences the ability of users to reduce the cost of capital, and make a profitable allocation of capital, investments, and/or loans.

Studying the quality of financial reports and observing their effect on the stock trading price movement was also conducted by Rashid (2020) by researching 296 annual reports concerning the years 2015 and 2016. Based on a sample of 125 companies between 2008 and 2017, the study by Asma et al. (2022) examines the relationship between the quality of financial reporting and the efficiency of investments made in the listed entity. Yidan (2021) also studied the relationship between initial reporting quality and investment efficiency. The research findings highlight the positive association between the two components.

Furthermore, based on a sample of companies across 76 countries, the study by Quoc et al. (2022) examines the relationship between the quality of financial reporting and dividend payout. The study underlines a stronger correlation between both elements in companies with high free cash flow problems and high asymmetry of information.

Another study documents the favorable economic consequences associated with mandatory IFRS adoption, where it is argued that IFRS adoption directly contributes to improving the quality of accounting information by contributing to improvements in analyst forecast accuracy and reduced dispersion, improvements in liquidity, and reduced cost of equity capital, positive price responses to events suggesting an increase in the likelihood of mandatory IFRS adoption (Wahyuni, 2020). We find that the results of this study are in contradiction with what is presented in a previous study Nulla (2014) namely, each country implements the same accounting standards differently, which causes a reduction in the quality of financial reports, concluding that IFRS adoption leads to an improvement in the quality of financial reports.

Based on a commitment and trust theory perspective, Avishek (2022), revealed higher financial reporting quality among the companies with higher buyback (share buyback) completion rates.

Also, using a sample of publicly listed firms in China, Hongkang (2022), documented the association between corporate site visits and financial reporting quality. Their research also identified that the association is more pronounced in firms with a higher degree of institutional ownership and state-owned enterprises.

### Instruments for measuring the quality of financial reporting

Various researchers have developed numerous methods to assess the quality of financial reporting, allowing the evaluation of financial reports based on qualitative characteristics of accounting information, such as Al-Dmour et al. (2018) and Kalyani et al. (2019). These methods differ both in terms of the content of the underlying assumptions and the form of implementation and the set of qualitative characteristics to be assessed. The lack of a common approach among accounting specialists on the formation of a methodology for assessing the quality of financial reporting is a consequence of different understanding of why a qualitative approach is needed in accounting and the essence of the concept of quality of accounting information and financial reporting (Plakhtiy, 2017).

A large part of the methods developed for assessing the quality of financial reporting is of the expert type, which provides for the consistent implementation of the following stages: structuring the qualitative characteristics of accounting information, determining the degree of compliance of its qualitative characteristics with reference indicators, calculating the basic indicator of accounting information quality, ranking the qualitative characteristics of information by users, and calculating the integrated indicator of accounting information quality (Pravdiuk et al., 2021).

To assess the quality of financial reporting, a variety of measurement, determination, and quantification methods have been used in previous research, such as:

– Accrual methods: (Bajra, 2018; Hoang, 2021). Being the most commonly used method, accruals methods are often linked with “accrual accounting,” which mandates the recognition of revenue at the time it is earned and expenses at the time they are incurred (Dănescu and Luminiţa, 2013).
– Methods that focus on the study of specific elements of annual reports: (Željana and Mario, 2021).
– Methods based on qualitative characteristics of information: (Yurisandi and Puspitasari, 2015; Mohd et al., 2016; Rashid, 2020).

## Materials and methods

Alternatives to help facilitate the visualization of bibliometric networks include VOSviewer. In the case of visualizations provided by VOSviewer, the distance between two nodes roughly suggests the relationship between those nodes. By providing distance-based rather than graph-based visualizations, VOSviewer is particularly suitable for visualizing large networks. Using the Web of Science database, we entered the following keywords to generate the article database used: financial reporting quality, information asymmetry, information asymmetry on the stock market, measuring financial reporting quality, and corporate responsibility. We also applied filters as the period of scientific research, 2005–2022, and the category to which the articles belong: Business, Business Finance, Management, and Economics. Following the application of these filters, we built a database of approximately 12,000 scientific articles, book chapters, and other scientific research.

To achieve the main objective of this research, we proceeded to the empirical evaluation of the quality of financial reports. In the research approach, we set subsequent objectives to identify possible effects of the COVID pandemic, on the quality of financial reports, as well as to identify possible discrepancies between the two markets of the Bucharest Stock Exchange, from a qualitative perspective.

We based the approach to quantify the quality of financial reports on the calculation of the quality measurement index developed by the NiCE. The index that underlies the validation or not of the formulated hypotheses we developed according to the qualitative characteristics of financial information established by the IASB, respectively, FASB, such as relevance and accurate representation, respectively, comparability, verifiability, timeliness, and understandability. The extended version of the index can be found in the first Supplementary Appendix.

The following hypotheses underline the research conducted:

(H1) There are no significant differences between the quality of financial reports of the two trading segments of the BVB, pre, and post-pandemic.

(H2) There is a negative correlation between the value of the quality measurement index and the following indicators: Capitalization, Price/Earnings ratio (P/Earnings), Price/Sales ratio, Price/Book Value ratio, Return on Equity (ROE), Return on Total Assets (ROA), respectively.

To form the first Hypothesis: **(H1) There are no significant differences between the quality of financial reports of the two trading segments of the BVB, pre and post-pandemic** we based on the method used by Suciati (2018) to measure the quality of Pre and Post IFRS financial reports. In contrast to Suciati’s (2018) research through research, we contribute to the literature by observing, calculating, and interpreting how the health crisis has impacted the quality of financial reports in the Romanian capital market.

The second Hypothesis: **(H2) There is a negative correlation between the value of the quality measurement index and the following indicators: Capitalization, Price/Earnings ratio (P/Earnings), Price/Sales ratio, Price/Book Value ratio, Return on Equity (ROE), Return on Total Assets (ROA), respectively,** was established taking into account the main financial indicators that investors are looking at when investing their capital. This hypothesis aims to draw attention to the correlation between a company’s financial indicators and the quality of its publicly available financial reports.

The research sample includes companies whose shares are traded on both the main and secondary segments of the Bucharest Stock Exchange (BVB). From the total number of traded companies, we have eliminated companies operating in the financial sector, those included in the monitoring list, those in insolvency proceedings, and those whose financial reports are not published on the BVB website. Thus, the sample includes 61 entities operating in the main segment of the BVB and 44 entities whose shares are traded in the secondary segment.

To study the macroeconomic influence generated by the health crisis, we included the research’s 2018–2021 financial reporting period. We considered 2018, a pre-pandemic year, 2019 and 2020 during the health crisis, followed by 2021, the first pre-pandemic year.

## Results

### Hypothesis testing

(H1) There is no significant difference between the quality of the financial reports of the two trading segments of the BVB, pre, and post-pandemic.

To verify the hypothesis, we performed a statistical test called the *t*-test, which is used to identify differences in certain data sets.

Table 1 includes the results obtained by applying the statistical test at the level of each quantitative characteristic to determine the degree of harmonization between the two regulated markets of the BVB during the periods studied. Affinities between the two categories of data are considered to exist when the result is below the value of 0.05. The results obtained above the value of 0.05 reinforce the hypothesis that there are significant differences in terms of the quality of financial reports between the two categories of segments.

**TABLE 1:** Statistical test results.

	2018			2019			2020			2021		
	pp	a	*T*-Test	pp	a	*T*-Test	pp	a	*T*-Test	pp	a	*T*-Test
R1	1.820	1.716	**0.220**	1.850	1.716	**0.208**	1.850	1.716	**0.194**	1.983	1.716	**0.174**
R2	2.016	1.506		2.050	1.506		2.083	1.506		2.167	1.506	
R3	3.492	4.000		3.500	4.000		3.533	4.000		3.483	4.000	
R4	3.000	1.914		3.033	1.951		3.033	1.951		3.117	1.951	
F1	1.852	1.716	**0.104**	1.867	1.716	**0.124**	1.867	1.716	**0.124**	1.917	1.716	**0.116**
F2	3.574	3.012		3.550	3.099		3.550	3.099		3.567	3.099	
F3	3.033	1.914		3.017	1.951		3.017	1.951		3.033	1.951	
F4	4.492	4.778		4.483	4.815		4.483	4.815		4.483	4.815	
F5	4.934	4.704		4.967	4.765		4.967	4.765		4.967	4.765	
U1	3.967	3.358	**0.019**	4.050	3.519	**0.022**	4.050	3.519	**0.022**	4.083	3.519	**0.021**
U2	4.918	3.802		4.950	3.889		4.950	3.889		4.950	3.889	
U3	4.902	3.827		4.917	3.938		4.917	3.938		4.917	3.938	
U4	4.410	4.000		4.417	4.086		4.417	4.086		4.417	4.086	
U5	1.000	1.000		1.000	1.000		1.000	1.000		1.000	1.000	
C1	3.557	3.037	**0.138**	3.550	3.099	**0.151**	3.550	3.099	**0.157**	3.567	3.099	**0.144**
C2	3.557	3.037		3.550	3.099		3.550	3.099		3.567	3.099	
C3	2.984	2.420		3.017	2.395		3.017	2.395		3.017	2.395	
C4	2.279	2.012		2.267	2.012		2.267	2.012		2.300	2.012	
C5	1.607	2.247		1.633	2.210		1.617	2.210		1.617	2.210	
C6	3.016	2.877		2.983	2.963		2.983	2.963		3.000	2.963	
T1	5.000	5.000	***N/A***	5.000	5.000	***N/A***	5.000	5.000	***N/A***	5.000	5.000	***N/A***

Own research.

The results obtained for each qualitative characteristic show disagreements in the following characteristics: Relevance (R), Accurate Representation (F), and Comparability (C), while no disagreements were found for the other characteristics.

The biggest differences are found in the fundamental qualitative characteristic of relevance, which is designed to help money holders make the best financial decision. It can be seen that within relevance, a decisive factor was held by R4, which answers the question: to what extent do the results provide feedback to users on how various market events and significant transactions have affected the company? To provide as objective a score as possible for this question, aspects such as: Whether or not there is a section in the annual report where significant market events and transactions are listed, whether or not explanations are given and not just a listing of existing events, but whether or not the effects of the reported events on the way the company conducted its business are explicitly presented. Thus, it appears that the financial reports of companies operating in the core segment are more relevant than those operating in the non-core segment.

The next qualitative characteristic, in terms of the resulting value, is Comparability (C), which is designed to help identify trends in a company’s performance and compare the information found with that of other similar companies. Significant differences in favor of the main segment are found in C1, which answers the question: To what extent do the explanatory notes on changes in accounting policies explicitly explain the causes and reasons for the changes that have occurred? and C2, which seeks to answer the question: To what extent do the explanatory notes on revisions to accounting estimates explicitly explain the causes and reasons for the changes that have occurred? The interpretation of the results obtained leads to the conclusion that the financial reports of companies in the main segment are more comparable than those of the other categories of companies in the secondary segment.

Significant differences are found in case F3, which seeks answers to the question: To what extent does the company highlight both positive and negative events in its annual results presentations?

The two qualitative characteristics of the financial reports, which tell that there are no differences between the two segments of the BVB are Intelligibility and Timeliness. In this respect, the results determine that the extent to which published reports are understood and timely by users is similar.

(H2) There is a negative correlation between the value of the quality measure index and the indicators: capitalization, price/earnings ratio (P/Earnings), price/sales ratio, price/book value ratio, return on equity (ROE) and return on total assets (ROA), respectively.

#### Main segment

An overall check on the statistical significance of the proposed model is qualified by the *F*-test (F-statistic, ANOVA table). With an F- Sig value below 1% (assumed risk threshold), the model demonstrates statistical significance, rejecting the null hypothesis on the relationship between the calculated index and the determined financial indicators. Therefore, the independent variable influences the dependent variable, and the results are statistically significant in terms of supporting the hypothesis. The summary of the indicators in the main market of the Bucharest Stock Exchange, along with the ANOVA test are presented in Tables 2, 3.

**TABLE 2:** Summary of indicators (main market).

	2018		2019		2020		2021	
	Mean	Std.	Mean	Std.	Mean	Std.	Mean	Std.
Index	3.3052	0.43940	3.3146	0.43668	3.3169	0.43859	3.3169	0.43859
Price_earnings	7.5089	13.73720	10.9693	16.33695	10.7303	20.02481	10.7303	20.02481
Price_sales	2.4325	3.84224	6.4769	23.27045	3.2451	6.01172	3.2451	6.01172
Price_bookValue	1.1480	1.92181	1.2000	2.01508	1.2236	1.80428	1.2236	1.80428
ROE	0.0615	0.42126	0.0772	0.20422	0.0630	0.17575	0.0630	0.17575
ROA	0.0385	0.12528	0.0466	0.11387	0.0134	0.10580	0.0134	0.10580

Authors’ projection.

**TABLE 3:** ANOVA (main market).

2018						2020					
	Sum of squares	df	Mean square	F	Sig.		Sum of squares	df	Mean square	F	Sig.
Regression	1.859	5	0.372	2.112	0.078^b^	Regression	2.651	5	0.530	2.243	0.063^b^
Residual	9.683	55	0.176			Residual	12.997	55	0.236		
Total	11.541	60				Total	15.648	60			

**2019**						**2021**					
Regression	2.255	5	0.451	1.852	0.118^b^	Regression	2.272	5	0.454	1.869	0.115^b^
Residual	13.393	55	0.244			Residual	13.375	55	0.243		
Total	15.648	60				Total	15.648	60			

Authors’ projection.

The Pearson correlation coefficient was applied to establish whether or not correlations exist between the index value obtained and the indicators initially established. Thus, Table 4 contains the quantitative assessment which measures both the direction and the intensity of the trends and correlations. The negative value obtained in the case of the price-earnings ratio determines that the volume of sales made by companies operating in the main segment does not directly influence the quality of the reports made available to users.

**TABLE 4:** Correlations (main market).

	Index	Price_earnings	Price_sales	Price_bookValue	ROE	ROA
2018	1.000	0.341	–0.176	0.322	0.149	0.261
2019	1.000	0.193	0.102	0.293	0.236	0.222
2020	1.000	0.168	–0.164	0.308	0.273	0.301
2021	1.000	0.168	–0.164	0.308	0.273	0.301

Authors’ projection.

In 2018, a slight to moderate correlation is observed in the case of the price/earnings ratio and the price/equity ratio, from which it is concluded that a higher value of these indicators leads to an increase in the quality of the financial reports, i.e., in the transparency of the company-investor relationship. The correlation between the index and the price-earnings ratio decreases over the years, especially in 2019, the first year associated with the Coronavirus pandemic. As regards the price-earnings ratio, the correlation between the ratio and the quality of the reports delivered to users remains on the same trend.

The indicator that measures the efficiency of the use of assets, in terms of the net result obtained, cannot be correlated with the pre-and post-pandemic period, but it can be observed that the increasing value of the correlation over the 4 years contributes directly to the quality of the financial reports.

#### AeRO segment

An overall check on the statistical significance of the proposed model is qualified by the *F*-test (F-statistic, ANOVA table). With an F- Sig value below 1% (assumed risk threshold), the model demonstrates statistical significance, rejecting the null hypothesis on the relationship between the calculated index and the determined financial indicators. Therefore, the independent variable influences the dependent variable, and the results are statistically significant in terms of supporting the hypothesis. The summary of the indicators in the AeRO segment, along with the ANOVA test are presented in Tables 5, 6.

**TABLE 5:** Summary of indicators (main market).

	2018		2019		2020		2021	
	Mean	Std.	Mean	Std.	Mean	Std.	Mean	Std.
Index	3.3169	0.43859	3.06170	0.51068	3.06170	0.51068	3.06170	0.51068
Price_earnings	10.7303	20.02481	13.83115	21.25992	21.74541	63.16811	9.16180	19.64756
Price_sales	3.2451	6.01172	3.76541	6.06407	3.94492	6.79140	4.09541	7.25538
Price_bookValue	1.2236	1.80428	2.15902	4.75498	2.89836	12.00023	1.27590	2.17286
ROE	0.0630	0.17575	–0.09131	1.22029	0.02344	0.32061	0.06098	0.19582
ROA	0.0134	0.10580	0.01574	0.17674	0.03246	0.16040	0.03246	0.09719

Authors’ projection.

**TABLE 6:** ANOVA (main market).

2018						2020					
	Sum of squares	df	Mean square	F	Sig.		Sum of squares	df	Mean square	F	Sig.
Regression	2.288	5	0.458	2.707	0.029	Regression	1.859	5	0.372	2.112	0.078
Residual	9.297	55	0.169			Residual	9.683	55	0.176		
Total	11.584	60				Total	11.541	60			

**2019**						**2021**					
Regression	2.023	5	0.405	2.363	0.052	Regression	1.859	5	0.372	2.112	0.078
Residual	9.418	55	0.171			Residual	9.683	55	0.176		
Total	11.441	60				Total	11.541	60			

Authors’ projection.

Using the same calculation method as for companies operating in the main segment of the BVB, Table 7 contains a quantitative assessment of the correlations between the index calculated based on the qualitative characteristics, i.e., the indicators initially established. Negative correlation values are more widespread in this case and are found in particular in the case of the price-income ratio, but also the case of the return on equity during the pandemic period. The correlation between the quality measure index and return on assets was also affected by the pandemic situation, registering lower values in the period 2019–2020.

**TABLE 7:** Correlations (AeRO market).

	Index	Price_earnings	Price_sales	Price_bookValue	ROE	ROA
2018	1.000	0.168	–0.164	0.308	0.273	0.301
2019	1.000	0.125	0.120	0.276	–0.054	0.124
2020	1.000	0.125	0.120	0.276	–0.054	0.124
2021	1.000	0.131	–0.002	0.267	0.349	0.335

Authors’ projection.

## Conclusion

**FIGURE 1:**
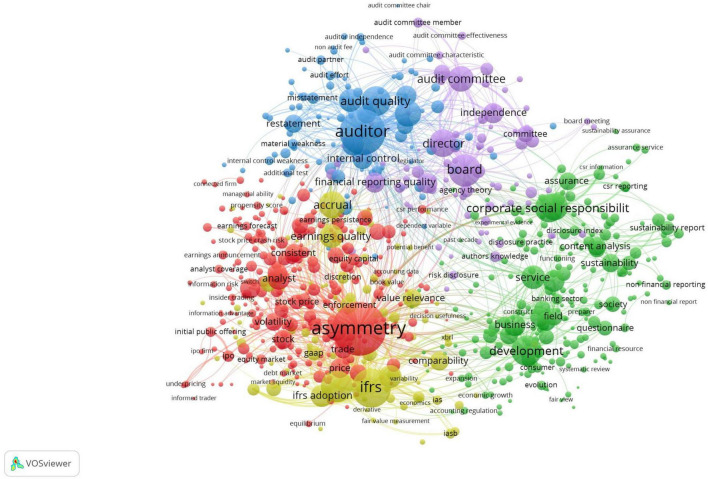
Bibliometric keyword analysis, based on VOSViewer.

In Figure 1 we have plotted the network resulting from the study of the relevant keywords in the title and abstracts. After mapping all the resulting words, we obtained several five clusters, i.e., 795 keywords that are part of them. It can be seen that the dominant topics or keywords were: asymmetry, auditor, IFRS, development, and corporate social responsibility, these being the most discussed topics by the researchers during the period analyzed. We consider that nodes or keywords that do not intertwine or network closely enough with other keywords have the potential to become new research topics in the future.

**FIGURE 2:**
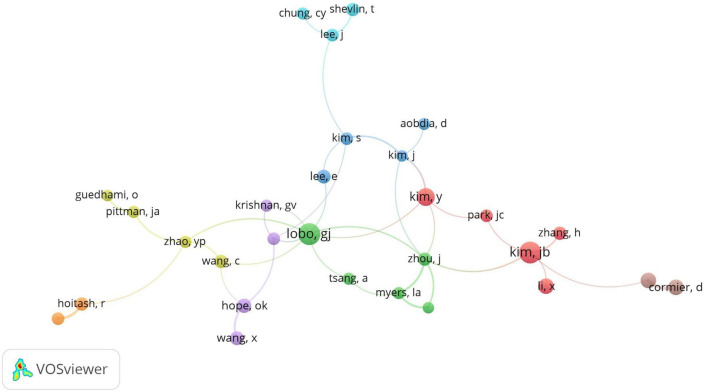
Bibliometric analysis of co-authorship, based on VOSViewer.

Collaborative scientific networks are a hallmark of contemporary academic research. Researchers are no longer independent actors, but members of teams bringing together complementary skills and multidisciplinary approaches around common goals. Social network analysis and co-authorship networks are increasingly used as powerful tools to assess collaborative trends. For this analysis, we considered a minimum of 5 co-authored papers. Following the selected options, we have compiled the co-authorships bibliometric analysis map, shown in Figure 2.

The main objective of this research derives from the desire to contribute to the diversification of the literature by identifying possible management responses in the area of financial reporting quality in the context of the COVID-19 pandemic event. Following the mapping of keywords and the realization of bibliometric structures, we observed that the dominant topics or keywords were: asymmetry, auditor, IFRS, development, and corporate social responsibility, these being the most discussed topics by researchers during the period under review, while scientific collaboration networks are increasingly emphasized in this research area.

Starting from the premise that the qualitative content of financial reports has as its starting point the decisions taken by management, making significant contributions to the investment process, in the first step we calculated the qualitative index of companies listed on the Bucharest Stock Exchange.

Since the requirements for admission to trading differ significantly for companies that pursue trading of shares on the main and secondary segments, and the behavior manifested is different, both by management and by users of financial information, we hypothesized that there would be qualitative differences between the financial reports of listed entities within the two categories of trading segments on the BVB, the main and the secondary. This hypothesis can be confirmed based on the qualitative characteristics of the financial reports: Relevance (R), Accurate Representation (F), and Comparability (C). The perspective determined by the detailed study of this hypothesis has in view for further research based on comparisons between the Romanian stock market and various other markets internationally.

From the perspective of the effects generated by the health crisis, we conclude that it has indirectly contributed to the divergence between the two BVB markets. It is observed that the year 2021 tends to eliminate the existing differences between the two markets, with the statistical indicator obtaining decreasing values during the 4 years studied. Also, in the period 2019–2020, the value of the indicators obtained at the level of each qualitative characteristic stagnates, resulting in a *T*-test value lying approximately at the same level.

The interpretation of the *t*-test results obtained leads to the conclusion that the financial reports of companies in the main segment are more comparable than those of the other categories of companies in the secondary segment.

Qualitative characteristics of financial information serve a major supporting role in the decision utility framework, specifically in the decision modeling approach in accounting theory. Qualitative characteristics are the attributes that make the information provided in financial statements useful to users. The same quality measurement index developed by NICE (Nijmegen Centre for Economics) was also used in Yurisandi and Puspitasari (2015) research. In contrast to the present research, his paper shows the following similarities and differences:

(1) The time period used is 2009–2010, while the period used in our research is longer (2018–2021), on which current political, economic and health events are reflected.
(2) The two studies use samples listed on different stock exchanges.
(3) Both researches conclude on the increase in the quality of financial information after major events, namely the adoption of IFRS in the case of Yurisandi and Puspitasari (2015) study and the current epidemiological situation, according to our research.

There are numerous researches that take qualitative characteristics as a starting point in measuring the quality of financial reporting, the most recent of which can be found in Table 8.

**TABLE 8:** Relevant studies.

Title	References	Sample
The impact of corporate governance regulations on board independence and quality of financial information reporting: A proposed study	Mohd et al., 2016	300 listed companies
Presence of professional accountant in the top management team and financial reporting quality Evidence from Bangladesh	Rashid, 2020	351 annual reports
Assessment of the financial reporting quality of South African and Indian listed companies	Haarburger, 2020	100 listed companies
Financial reporting quality and share price movement-evidence from listed companies in Bangladesh	Rashid, 2020	296 annual reports

Authors’ projection.

The differences between this research and those listed are the sample size, the country of origin of the companies, the period over which the research is conducted, and the associated secondary theme. While our paper considers the aspect related to the epidemiological situation, Mohd et al.’s (2016) study associates the quality of financial reporting with corporate governance and Rashid (2020) correlates this team with the presence of the accounting professional in the senior management team.

The existence of a behavioral reaction to the possible impact of the epidemiological situation on the quality of financial reports was also studied, considering that external factors have a significant contribution to the way of working, i.e., on the decisions taken by management. In this respect, four main observations were identified regarding the dynamics of the quality of financial reports during the 4 years studied:

(1) We identified four companies operating in the manufacturing sector and one company in the real estate sector that showed a positive response to the changes brought about by the pandemic, significantly improving the quality of their financial reports in the post-pandemic year, including the manner of reporting and the diversity of information contained in the report. This leads to better transparency and communication between companies and end users;
(2) We identified only one company whose financial reporting quality was significantly lower in 2021 than the reports published during the health crisis, which operates in the engine and turbine manufacturing sector. In this regard, we conclude that the external event represented by the health crisis negatively influenced the management’s reaction, contributing to a decrease in the quality of published reports;
(3) The financial reports of most of the entities in the sample (a total of 64 companies) showed consistency over the period analyzed, including in the number of pages, layout, and information contained, with the health crisis not influencing the way management chooses to provide financial information to end users;
(4) Companies were identified that did not report briefly or at all on negative events closely related to the epidemiological situation, including the impact on business continuity. This trend was observed in the case of small but long-established companies;
(5) In the post-pandemic period, most companies are paying more attention to the non-financial reporting section, mainly due to the increasing trend of amplifying and highlighting these categories of information, with a strong emphasis on the social component of economics.

The second hypothesis aims to determine which elements could directly influence the value of the calculated index. Thus, following correlations between the value obtained and indicators such as capitalization, price-earnings ratio, and price-income ratio, it is concluded that a slight to moderate correlation can be established in terms of the price-earnings ratio in the case of entities in the main segment, and a positive correlation between the index used and the capitalization of companies in the case of the second segment.

This approach is associated with the main limitation, which is the lack of financial reports of some issuers (13) on www.bvb.ro, although there are reporting requirements included. Another possible limitation associated with the primary objective is the origin of the quality measurement index that was considered, namely the Netherlands, due to the differences between the level of development of the countries of origin of the index and the level of development of the Romanian stock market. In this sense, another future perspective of this research aims at studying these existing differences and adapting the index to the characteristics of the Bucharest Stock Exchange.

## Author contributions

All authors listed have made a substantial, direct, and intellectual contribution to the work, and approved it for publication.
