# Supplementary material for: Companies’ behavior in measuring the quality of financial reports: Pre- and post-pandemic research
Source: Front Psychol. 2022 Dec 19;13:1005941. doi: 10.3389/fpsyg.2022.1005941 (PMC9807020; doi:10.3389/fpsyg.2022.1005941)
Supplement: Supplementary file 1 [file Data_Sheet_1.docx]

| No. crt | **Question** | **Rating System** |  |
| --- | --- | --- | --- |
| **Relevance** | | |  |
| **RI** | To what extent does the presence of the outlook statement contribute to the formation of expectations and forecasts about the future of the entity? | 1= no forecast information; 2= predictive information is not a separate subsection; 3= separate subsections; 4= extensive forecast; 5= extended predictions useful to create expectations. |  |
| **R2** | To what extent does the presence of non-financial information complement financial information? | 1= no non-financial information; 2= little non-financial information, not useful for expectation formation; 3= useful non-financial information; 4= non-financial information is useful for developing expectations. |  |
| **R3** | To what extent does the company use fair value rather than historical cost? | 1= Historical cost only (HC); 2= Mostly HC; 3= Balance between Fair Value (FV)/HC; 4= Largest share of FV; 5= FV only |  |
| **R4** | To what extent do the results presented provide feedback to users of annual reports on how various market events and significant transactions have affected the entity? | 1= No feedback; 2= Little feedback about the past;  3= Feedback is present; 4= Feedback helps to understand how events and transactions have influenced the company; 5= Comprehensive feedback |  |
| **Accurate representation** | | |  |
| **Fl** | To what extent are valid arguments provided to support the decision for certain assumptions and estimates in the annual report? | 1= Described estimates only;  2= General explanations; 3= Specific explanations of estimates; 4= Specific explanations, formulas explained;  5= Comprehensive argumentation. |  |
| **F2** | To what extent does the company base its choice of accounting policies on valid arguments? | 1= Unexplained changes; 2= Minimal explanation; 3= Reason explanation; 4= Reason + consequences explanation; 5= Comprehensive explanation. |  |
| **F3** | To what extent does the company highlight both positive and negative events in the discussion of annual results? | 1= Negative events mentioned only in footnotes;  2= Emphasis on positive events; 3= Emphasis on positive events, but negative events are also mentioned; 4= Balances positive/negative events; 5= Impact of both events is explained |  |
| **F4** | What type of audit opinion is included in the annual report? | 1= Contrary opinion; 2= Unable to express an opinion; 3= Opinion with reservations; 4= Unqualified opinion; 5= Unqualified opinion: Financia figures + internal control |  |
| **F5** | To what extent does the company provide information on corporate governance | 1= No description of corporate governance;  2= Limited information, separate subsection;  3= Separate subsection 4= Increased attention paid; |  |
|  |  | 5= Comprehensive description |  |
| **Intelligibility** | | |  |
| U I | To what extent is the annual report presented in a well-organized manner? | Reasoning based on: typology of tables; name of section; order of sections; summary/conclusion of each subsection |  |
| U2 | To what extent are the explanatory notes presented clearly enough? | 1= No explanation; 2= Very short description, difficult to understand;  3= Explanations describing events; 4= Explanation of terms is present; 5= Everything that might be difficult to understand is explained |  |
| U3 | To what extent does the presence of graphs and tables clarify the information presented? | 1= No graphs; 2= 1-2 graphs; 3=3-5 graphs; 4=6- 10 graphs; 5=>5 graphs |  |
| U4 | How easy is it to follow the use of language and technical reasoning? | 1= A lot of unexplained jargon; 2= Much jargon, very little explanation  3= Minimal explanations; 4= Minimal jargon; 5= No or very well-explained jargon |  |
| U5 | What is the size of the glossary? | 1= Glossary not available; 2= Glossary less than one page;  3= approximately one page; 4= 1-2 pages; 5=>2 pages |  |
| **Comparability** | | |  |
| Cl | To what extent do the explanatory notes on changes in accounting policies explain information about the change in accounting policies? | 1= Unexplained changes; 2= Minimal explanations;  3= Explanation of reasons; 4= Explanation of reasons and consequences; 5= No changes or comprehensive explanation of changes |  |
| C2 | To what extent do the explanatory notes on revisions to estimates explain information on changes in estimates? | 1=Reviews without explanations; 2= Revisions with minimal explanation;  3= No transparent revisions/explanations;  4= Transparent notes + explanations; 5= Comprehensive explanations. |  |
| C3 | To what extent has the company adjusted prior period figures for the effect of implementing a change in accounting policy or revisions to accounting estimates? | 1=No adjustments; 2= Adjustments described; 3= Adjustments for the current period only;  4=Adjustments for 2 years; 5=>2 years + explanatory notes |  |
| C4 | To what extent does the entity provide a comparison of the current accounting period with the previous accounting period? | 1= No comparisons; 2= Only compared to the previous year;  3= Comparisons with 2-5 years; 4= 5 years of comparisons 5= 10 years of comparisons + description of implications |  |
| *C5* | To what extent is the information in the annual report comparable with the information provided by other organizations? | Judgement based on: -accounting policies; -structure - explanations of events |  |
| C6 | To what extent does the company present financial figures and indicators in its annual reports? | 1= No indicators; 2= 1-2 indicators; 3= 3-5 indicators; 4= 6-10 indicators; 5=> 10 indicators |  |
| **Opportunity** | | |  |
| Tl | How many days were needed for the auditor to sign the audit report after the end of the financial year | 1=1-1,99; 2=2-2,99; 3=3-3,99; 4=4-4,99; 5=5-5,99 |  |
